# The Effect of Preoperative Information and Education on the Clinical Outcome of Total Hip Arthroplasty: A Prospective, Randomized Trial

**DOI:** 10.7759/cureus.73841

**Published:** 2024-11-17

**Authors:** Sara Eleni Amprachim, John Vlamis, Melina J Vlami, Vasileios S Nikolaou, Spyros G Pneumaticos

**Affiliations:** 1 3rd Department of Orthopaedics, National and Kapodistrian University of Athens School of Medicine, KAT Attica General Hospital, Athens, GRC; 2 2nd Department of Orthopaedics, National and Kapodistrian University of Athens School of Medicine, Athens, GRC

**Keywords:** clinical outcome, duration of hospitalization, preoperative education, preoperative information, total hip arthroplasty

## Abstract

Introduction

Preoperative patient information and education is an essential aspect of modern surgical care, particularly for patients undergoing total hip arthroplasty (THA). This prospective, randomized trial aimed to assess the effects of structured preoperative education and information on clinical outcomes in patients undergoing THA.

Materials and methods

A total of 102 patients were randomized into two groups: the intervention group (n = 51) receiving standardized preoperative information and education, and the control group (n = 51) receiving standard preoperative care without a formal educational component. Postoperative outcomes, including functionality, mobility, length of hospital stay (LOS), patient satisfaction, health-related quality of life, anxiety, depression and fear for surgery, were compared between the two groups.

Results

Both groups were comparable in baseline characteristics, including age, sex, body mass index (BMI), smoking status and alcohol consumption. The mean age was 66.3 years, mean BMI was 29.05 and 70.6% of participants were female. Patients in the intervention group had a shorter mean hospital stay (mean 4.9 days vs. 6.2 days, p=0.031). Mean preoperative modified Harris Hip Score (mHHS) was similar between the two groups (p = 0.866). However, one month postoperatively, mHHS was significantly higher in the intervention group compared to controls (74.06 versus 67.81, p = 0.046). The absolute change in European Quality of Life 5 Dimensions 5 Level (EQ-5D-5L) Index and EQ-5D-5L Visual Analogue Scale (VAS) score before and after THA was statistically significant (p = 0.021 and p = 0.042). Preoperative and postoperative depression was significantly lower in the intervention group, one day preoperatively, one day before discharge and one month postoperatively, as shown by the Hospital Anxiety and Depression Scale (HADS) (p = 0.026, p = 0.027 and p = 0.018 respectively).

Conclusions

This prospective, randomized trial demonstrated that preoperative education and information significantly improve clinical outcomes, duration of hospitalization, health-related quality of life and postoperative anxiety in patients undergoing THA. These findings underline the importance of incorporating structured educational programs into preoperative care protocols prior to THA for enhancing patient recovery and optimizing postoperative results.

## Introduction

Osteoarthritis (OA) is the most common joint disorder in the world [1]. An estimated 31 million adults in the United States and 300 million people worldwide live with OA, which is the leading cause of disability in older adults and leads to pain, loss of function, and poor quality of life [2]. Hip OA is one of the most common types of osteoarthritis affecting millions of patients globally. Total hip arthroplasty (THA) is one of the most successful surgical interventions for treating end-stage hip osteoarthritis and other degenerative joint diseases. The procedure has proven to significantly alleviate pain, restore mobility, and improve quality of life in individuals suffering from debilitating hip conditions. However, despite the advances in surgical techniques and biomaterials, THA is a major surgery that involves significant physical and psychological stress and expectations and satisfaction remain unfulfilled for a plethora of patients [3].

One critical factor in optimizing clinical outcomes in THA is the provision of preoperative information and education to patients. Preoperative education is defined as “any educational intervention provided before surgery that aims to improve people's knowledge, behaviors, and health outcomes” [4]. Preoperative patient education and information have been recognized as an essential component of rehabilitation programs [5]. Preoperative information and education may include surgical steps, type of anesthesia, potential risks and complications, hospital discharge planning, expected outcomes, post-operative pain management and early recovery programs when indicated [5,6]. By providing patients with a clear understanding of the surgery and the recovery process, preoperative education can reduce anxiety, enhance patient engagement, and improve adherence to postoperative care protocols. Furthermore, educating patients about potential complications such as infections, venous thromboembolism, and prosthesis dislocation can promote early recognition and timely intervention [7].

Several studies have demonstrated the benefits of preoperative education across various surgical procedures, suggesting improvements in patient satisfaction, reduced postoperative pain, and shorter hospital stays. However, the specific impact of structured preoperative education on clinical outcomes in THA remains underexplored. Most available studies lack robust prospective designs and often fail to rigorously compare outcomes between patients who receive standardized education and those who do not [8]. Consequently, there is a need for high-quality research to assess whether preoperative education can tangibly improve postoperative recovery, functional outcomes, and complication rates in THA patients.

Taking into consideration the beneficial role of preoperative information and education, we have hypothesized that patients receiving structured preoperative education before THA will experience improved clinical outcome, shorter hospital stays, reduced fear and anxiety for the surgery and a better quality of life. The primary objective of this prospective, randomized trial is to evaluate the effect of preoperative information and education on the clinical outcomes and the psychological impact on patients undergoing THA.

## Materials and methods

Study design

**Figure 1:**
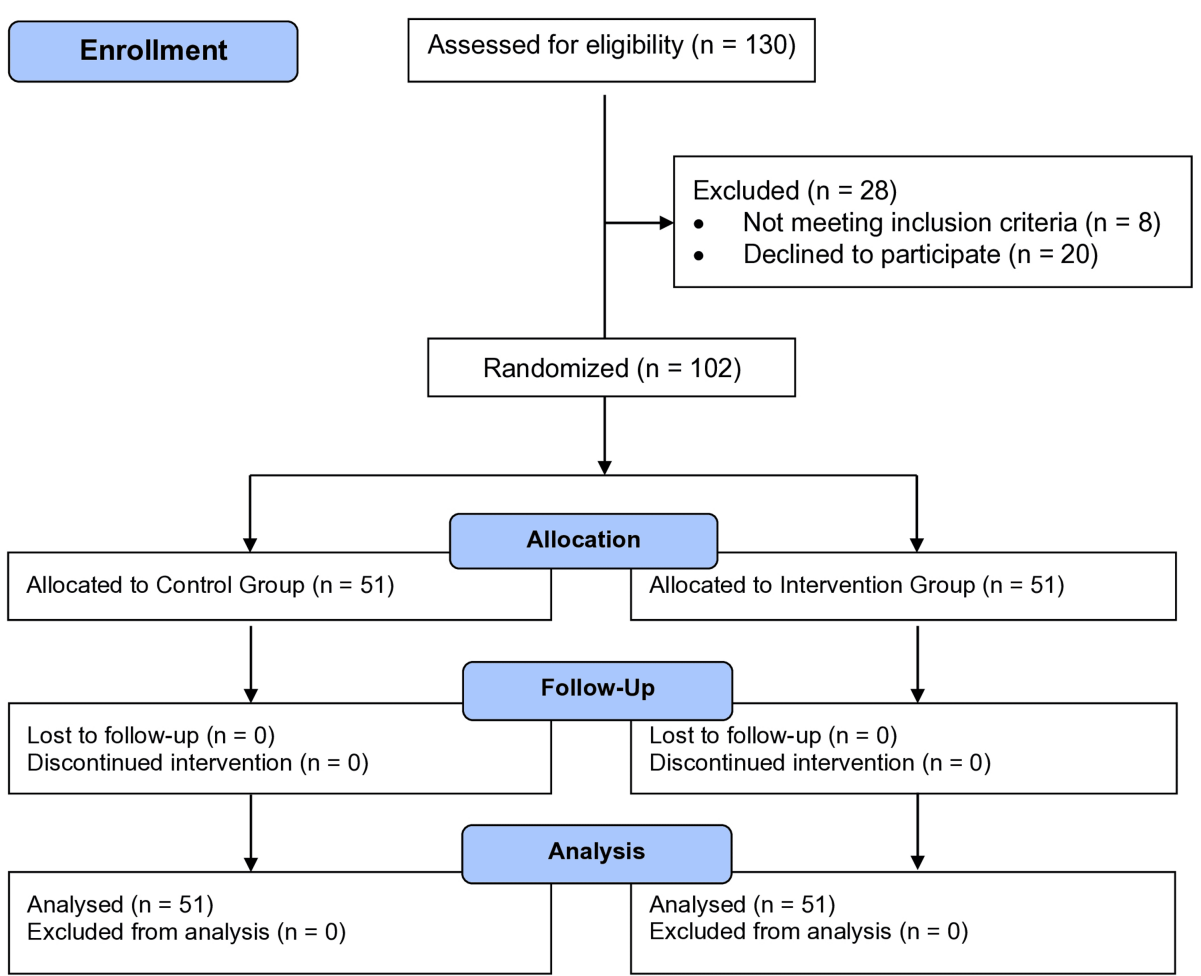
Flowchart of patients included in the study according to the Consolidated Standards of Reporting Trials (CONSORT) statement

This prospective, randomized trial was conducted between October 2022 and May 2024 at KAT Hospital, Athens, Greece (ClinicalTrials NCT06662981). The study flowchart according to the Consolidated Standards of Reporting Trials (CONSORT) recommendations is shown in Figure 1 [9].

Ethical approval was obtained from the institutional review board (approval 667/19.09.2022), and all participants provided informed consent prior to enrollment. Inclusion criteria were patients aged > 50 years, diagnosed with hip OA, and indicated for elective THA. All patients were operated by the same surgical team, with a posterior approach and placement of uncemented THA components. Exclusion criteria were age < 50 years, cognitive impairment and mental disorder.

A total of 102 patients scheduled for elective primary THA were recruited and randomized into two groups: Intervention Group (Group A): 51 patients received standardized preoperative information and education, including verbal instructions, and written material; and Control Group (Group B): 51 patients received the standard preoperative care, which did not include formalized education beyond routine consent.

Intervention

The intervention included oral and written education about the operation, nursing and active participation in self-care. Patients were encouraged to ask questions at any stage of the session and there was time for further discussion and questions. The intervention took place in three phases: one week before THA, one day before THA after patient admission and postoperatively, before patient discharge. Information and training were given orally and with written instructions to the intervention group, by the researcher or the attending physician. The instruction sheet was written in simple and understandable language and contained photographs that helped to understand the information provided.

Preoperative information, lasting 20 minutes, took place one week before THA so that any question that concerns the patient can be formulated. After a rough description of the operation, the anatomy and functionality of the joint were presented so that the patient understood the necessity of the operation. The necessity of preoperative examinations along with potential need for blood transfusion was discussed. Instructions were given for recognizing and managing potential complications (e.g., infection, dislocation, deep vein thrombosis). Patients were informed about the appropriate post-operative walking aids, walking style, sitting-up techniques, standing-up techniques, lying-down techniques and getting in and out of the car. In addition, there was a demonstration of muscle strengthening exercises, isometric quadriceps and gluteal exercises, triple flexion exercises, short-range knee extension, hip abduction and adduction, knee extension, pelvic lift and chair push-up. Patients were also informed about the necessity of prophylactic postoperative antithrombotic and antibiotic treatment. The need to prepare the house was discussed (preparation of the bathroom, kitchen, sleeping area).

On admission and before surgery, in the intervention group, pre-operative education and information was repeated. After surgery and before the patient discharge, in the intervention group, the instructions regarding the limitations of the operation were repeated. Patients were re-informed about effective pain management, the use of ice packs and the safe entry and exit of the car, while the presented exercises were performed in the presence of the researcher and the patients were encouraged to repeat them at regular intervals.

Outcomes measured

Patient demographic data and baseline clinical characteristics (gender, age, nationality, education level, work activity, marital status, weight, height, body mass index, comorbidities, smoking) were also recorded.

Clinical Parameters Assessed

Hip pain and functionality: Assessed using modified Harris Hip Score (mHHS), validated in Greek, one day preoperatively and one month postoperatively [10].

Mobility: Assessed using the Timed Up and Go (TUG) test, one day preoperatively [11].

Length of hospital stay (LOS): Measured in days and recorded from the time of surgery to discharge.

Anxiety and depression: Assessed using the Hospital Anxiety and Depression Scale (HADS), validated in Greek, one day preoperatively, one day before discharge from hospital, and one month postoperatively. It consists of two subscales: HADS-A assesses symptoms of anxiety and HADS-D assesses symptoms of depression [12].

Fear for surgery: Assessed using the Surgical Fear Questionnaire (SFQ), one day before THA [13].

Health-related quality of life: Assessed using EQ-5D-5L index and EQ-5D-5L visual analog scale (VAS), validated in Greek one day preoperatively, one day before discharge from hospital, and one month postoperatively [14].

Patients’ satisfaction about the provided information and education concerning their condition and the relevant operation: Assessed using Information Satisfaction Questionnaire (ISQ) one day before THA [15].

Risk of complications: Assessed using the Risk Assessment and Prediction Tool (RAPT) one day before THA [16].

Statistical analysis

The normality of the distributions was tested using the Kolmogorov-Smirnov test. The values ​​of the quantitative variables are presented using the mean and standard deviation (SD) in the case of a normal distribution as well as the median and the interquartile range (IQR) in the case of a non-normal distribution. The frequencies (ν) and the corresponding percentages (%) are used in the categorical variables. The comparison between patients in intervention group and patients in control group for the quantitative and qualitative variables was performed with the t-test for independent samples in case of normal distribution or the Mann-Whitney test in case of non-normal distribution and the x2 test respectively. All statistical analyzes were performed with the statistical package SPSS 17.00 (IBM Corporation, Armonk, NY, USA). All tests are two-sided. A p-value < 0.05 was defined as the level of statistically significant difference.

## Results

Demographic characteristics of the study population are shown in Table 1.

**Table 1:** Demographic characteristics of the study population SD: Standard Deviation BMI: Body Mass Index *: Chi-squared test **: Student's t-test

	Control Group	Intervention Group	Total	p-value *
	N (%)	N (%)	N (%)	
Gender				0.515
Men	13 (25.5%)	17 (33.3%)	30 (29.4%)	
Women	38 (74.5%)	34 (66.6%)	72 (70.6%)	
Married	34 (66.7%)	28 (54.9%)	62 (60.8%)	0.549
Lonely Living	9 (17.6%)	14 (27.4%)	23 (22.5%)	0.343
Public Insurance	48 (94.1%)	49 (96.1%)	97 (95.1%)	0.607
Smoking	11 (21.6%)	20 (39.2%)	31 (30.4%)	0.084
	Mean (SD)	Mean (SD)	Mean (SD)	p-value **
Age (years)	67.31 (7.77)	65.24 (8.71)	66.27 (8.28)	0.207
Weight (kg)	80.00 (13.89)	77.82 (13.84)	78.91 (13.85)	0.430
Height (cm)	164.51 (8.45)	165.24 (9.04)	164.87 (8.71)	0.676
BMI (kg/m^2^)	29.61 (4.88)	28.49 (4.59)	29.05 (4.74)	0.236

Both groups were comparable in baseline characteristics, including age, sex, BMI and smoking status and alcohol consumption. The mean age was 66.3 years, mean BMI was 29.05 and 70.6% of participants were female. 80.4% of participants were of Greek ethnicity. The majority of patients were retired, married, with children, lived at cities and had public insurance. The type of anesthesia was similar between the two groups (p = 0.120). Preoperative and postoperative clinical data are shown in Tables 2, 3. Table 4 shows the changes of the clinical scores between preoperative and postoperative values.

**Table 2:** Preoperative clinical variables of the study population, one day prior to surgery SD: Standard Deviation TUG: Timed Up and Go HADS: Hospital Anxiety and Depression Scale ISQ: Information Satisfaction Questionnaire SFQ: Surgical Fear Questionnaire mHHS: Modified Harris Hip Score VAS: Visual Analog Scale EQ-5D-5L: European Quality of Life 5 Dimensions 5 Levels RAPT: Risk Assessment and Prediction Tool *: Student's t-test. P-values adjusted for multiple testing.

	Control Group	Intervention Group	Total	
	Mean (SD)	Mean (SD)	Mean (SD)	p-value *
TUG TEST	16.51 (4.67)	17.00 (6.70)	16.75 (5.75)	0.900
HADS-A	6.12 (3.77)	6.22 (4.10)	6.17 (3.92)	0.900
HADS-D	6.06 (3.17)	4.75 (2.68)	5.40 (2.99)	0.286
ISQ	13.02 (5.03)	11.88 (4.63)	12.45 (4.85)	0.732
SFQ-short	14.04 (12.07)	16.63 (11.31)	15.33 (11.71)	0.732
SFQ-long	6.61 (9.03)	7.06 (8.21)	6.83 (8.59)	0.900
SFQ-total	20.65 (18.87)	23.69 (16.09)	22.17 (17.52)	0.762
mHHS	42.61 (13.05)	43.02 (11.58)	42.81 (12.28)	0.900
EQ-5D-5L INDEX	0.29 (0.23)	0.24 (0.17)	0.26 (0.21)	0.732
EQ-5D-5L VAS	65.69 (17.03)	63.18 (19.07)	64.43 (18.04)	0.762
RAPT	8.27 (2.24)	8.59 (2.17)	8.43 (2.19)	0.762

**Table 3:** Postoperative clinical variables of the study population SD: Standard Deviation LOS: Length of Stay mHHS: Modified Harris Hip Score HADS: Hospital Anxiety and Depression Scale EQ-5D-5L: European Quality of Life 5 Dimensions 5 Levels VAS: Visual Analogue Scale *: Student's t-test

	Control Group	Intervention Group	Total	
	Mean (SD)	Mean (SD)	Mean (SD)	p-value *
LOS (days)	6.24 (3.87)	4.94 (1.72)	5.59 (3.05)	0.032
Postoperative Values (1 day before discharge from hospital)				
HADS-A	5.41 (3.84)	4.94 (3.56)	5.18 (3.69)	0.523
HADS-D	5.76 (3.42)	4.31 (3.08)	5.04 (3.32)	0.027
Postoperative Values (1 month after THA)				
HADS-A	5.10 (3.49)	4.20 (3.80)	4.65 (3.66)	0.214
HADS-D	4.98 (3.22)	3.53 (2.87)	4.25 (3.12)	0.018
mHHS	67.81 (16.67)	74.06 (14.44)	70.94 (15.84)	0.046
EQ-5D-5L INDEX	0.53 (0.26)	0.60 (0.25)	0.57 (0.26)	0.187
EQ-5D-5L VAS	77.25 (13.50)	81.96 (11.75)	79.61 (12.81)	0.063

**Table 4:** Changes of clinical variables of the study population, defined as the difference between postoperative and preoperative values SD: Standard Deviation mHHS: Modified Harris Hip Score HADS: Hospital Anxiety and Depression Scale EQ-5D-5L: European Quality of Life 5 Dimensions 5 Levels VAS: Visual Analogue Scale *: Student's t-test

	Control Group	Intervention Group	Total	
	Mean (SD)	Mean (SD)	Mean (SD)	p-value *
Change of HADS-A	-1.02 (3.37)	-2.02 (4.15)	-1.52 (3.80)	0.185
Change of HADS-D	-1.08 (3.80)	-1.22 (2.30)	-1.15 (3.13)	0.826
Change of mHHS	25.25 (18.97)	31.04 (15.09)	28.15 (17.30)	0.091
Change of EQ-5D-5L INDEX	0.24 (0.26)	0.36 (0.24)	0.30 (0.26)	0.021
Change of EQ-5D-5L VAS	11.57 (16.72)	18.78 (18.54)	15.18 (17.94)	0.042

No significant differences in preoperative mobility between the two groups were detected in the TUG test (p = 0.669). Mean preoperative mHHS was similar between the two groups (p = 0.866). However, one month postoperatively, mHHS was significantly higher in the intervention group compared to controls (74.06 versus 67.81, p = 0.046). The absolute change in mHHS before and after THA was not statistically significant (p = 0.091). Preoperative patient satisfaction, as depicted in ISQ score, was similar between the two groups (p = 0.238). Similarly, no significant differences in risk of complications, as shown by the RAPT score, were detected (p = 0.423). Preoperative health-related quality of life was similar in the two groups (p = 0.225 and p = 0.485). One month after surgery, health-related quality of life was higher in the intervention group, with marginal statistical significance (p = 0.187 and p = 0.063). The absolute change in EQ-5D-5L Index and EQ-5D-5L VAS score before and after THA was statistically significant (p = 0.021 and p = 0.042), suggesting that the intervention led to postoperative improvement in health-related quality of life.

There were no differences in preoperative and postoperative anxiety, as depicted by the HADS-A (p = 0.900 and 0.214). Preoperative and postoperative depression was significantly lower in the intervention group, one day preoperatively, one day before discharge and one month postoperatively, as shown by the HADS-D (p = 0.026, p = 0.027 and p = 0.018 respectively). However, the absolute change in HADS-D before and after THA was not statistically significant (p = 0.826). Preoperative fear of surgery, as shown by the SFQ score was similar between the two groups (p > 0.05). Patients in the intervention group had a shorter mean hospital stay (mean 4.9 days vs. 6.2 days, p=0.031).

## Discussion

The results of this study support the hypothesis that structured preoperative education and information positively influence clinical outcomes in patients undergoing THA. Patients in the intervention group had higher mHHS scores, shorter hospital stays, significant improvement in health-related quality of life and experienced less preoperative and postoperative depression.

Preoperative information and education for THA covered several key components. First, patients were provided with an overview of the surgery, including a basic explanation of the purposes of the surgery, the basic techniques and the expected outcome. Patients were informed about the necessary preoperative medical evaluations to ensure they were fit for surgery. Medication management was also discussed, including the preoperative cessation of anticoagulants or lifestyle adjustments, such as quitting smoking, managing weight, and improving physical fitness. Patients were also educated about the details on the day of surgery, such as the fasting requirements, the types of anesthesia used, the approximate duration of the procedure and the expected length of the hospital stay [17]. Setting realistic expectations regarding postoperative pain is essential, so patients were warned about the anticipated pain levels and how it should decrease over time, along with the proper use of analgesics. In terms of rehabilitation, patients were informed about their physiotherapy and specific exercises to regain strength and function in the hip, along with the use of assistive devices like crutches, walkers, or canes [18]. Finally, patients were educated to early recognize potential THA complications [19].

Preoperative information and education for THA can be delivered through a variety of methods, such as one-on-one consultations with healthcare providers, preoperative classes or group education sessions, printed educational materials, multimedia tools, internet resources or mobile applications [20-22]. In the present study, we chose personalized discussions tailored to the patient’s specific medical condition and concerns, providing an opportunity for patients to ask questions and receive individualized advice. Additionally we used a booklet containing detailed explanations of the surgical process, postoperative care, and rehabilitation exercises. We chose to avoid multimedia tools or mobile applications, as most patients were above 60 and may not be familiar with the technological resources.

The results of the present study have shown that preoperative education and information have a positive impact on postoperative pain and functionality, as reflected by the increase of mHHS in the intervention group. This can be explained by the fact that preoperative education improves patient understanding of rehabilitation exercises, leading to better adherence to postoperative physiotherapy protocols and quicker recovery of mobility and strength. Moreover, preoperative education helps set realistic expectations about postoperative pain and recovery, which can lead to better psychological preparedness and improved pain tolerance.

Most studies have confirmed that preoperative patients’ information and education before THA led to a significant decrease in LOS [20,23-26]. This reduction of the duration of hospitalization has been estimated at 0.6 - 0.8 days [20,25,26]. In the present study, preoperative education and information have been associated with a mean 1.3 days reduction in LOS. This positive impact may be attributed to the beneficial effects of preoperative education and information in terms of faster recovery, better pain management and better control of postoperative complications.

Fear of surgery, anxiety and depression prior to THA, may increase pain sensitivity and impair patients’ ability to deal with pain [27]. The results of the present study showed that preoperative education and information have a minimal impact on fear for surgery and anxiety, both preoperatively and postoperatively. Literature data have shown contradictory results, as there are many studies supporting the beneficial role of preoperative education and information on anxiety [4,17,28], while other studies found no significant association with anxiety reduction [23,29]. It has been suggested that the effect of preoperative information on anxiety depends on the type of education technique and the education provider [23,28]. On the other hand, in the intervention group, lower scores of depression were noted, before and after THA. Similarly, in a study by Huang et al, a preoperative educational program resulted in lower depressive inclinations in patients undergoing THA [30].

Preoperative information and education before THA may significantly enhance health-related quality of life by improving physical function, pain management, mental health, and overall satisfaction with the surgical outcome. The results of the present study have shown that the intervention group experienced higher changes in EQ-5D scores compared to controls, meaning that preoperative information and education led to improved health-related quality of life. By reducing anxiety, promoting faster recovery, and empowering patients to take an active role in their rehabilitation, preoperative education ensures that patients enjoy a higher quality of life after surgery.

The present study encompasses several limitations. First, while the sample size is relatively sufficient, the follow-up period of one month is somehow short, raising the possibility that the long-term effects of the intervention may not be captured. The study may be single-blinded as the data were processed by a different person than the data collector, but it could not be double-blinded as patients were aware of the education provided. This could lead to performance bias, where participants in the intervention group are more motivated or report better outcomes due to their knowledge, rather than the actual effect of the intervention. Moreover, the degree of participants' adherence to the educational recommendations may vary. Some patients may engage fully with the preoperative information and rehabilitation plans, while others may not, leading to differences in outcomes that are not entirely attributable to the intervention itself. This variability could dilute the apparent effect of the preoperative information.

## Conclusions

This prospective, randomized trial demonstrated that preoperative education and information significantly improve clinical outcomes, duration of hospitalization, health-related quality of life and postoperative anxiety in patients undergoing THA. These findings underline the importance of incorporating structured educational programs into preoperative care protocols prior to THA for enhancing patient recovery and optimizing postoperative results. Future studies with longer follow-up periods and larger sample sizes may further elucidate the long-term benefits of preoperative education and information on THA outcomes.
